# Impact of weight changes on the incidence of diabetes mellitus: a Korean nationwide cohort study

**DOI:** 10.1038/s41598-018-21550-3

**Published:** 2018-02-27

**Authors:** Eun Sook Kim, Jee Sun Jeong, Kyungdo Han, Mee Kyoung Kim, Seung-Hwan Lee, Yong-Moon Park, Ki Hyun Baek, Sung Dae Moon, Je-Ho Han, Ki-Ho Song, Hyuk-Sang Kwon

**Affiliations:** 1 Department of Internal Medicine, College of Medicine, The Catholic University of Korea, Seoul, 06591 Korea; 2 Department of Biostatistics, College of Medicine, The Catholic University of Korea, Seoul, 06591 Korea; 3 Epidemiology Branch, National Institute of Environmental Health Sciences, National Institutes of Health, Research Triangle Park, NC, 27709 USA; 4 Division of Endocrinology and Metabolism, Department of Internal Medicine, Incheon St. Mary’s hospital, Incheon, 21431 Korea; 5 Division of Endocrinology and Metabolism, Department of Internal Medicine, Yeouido St. Mary’s hospital, Seoul, 07345 Korea; 6 Division of Endocrinology and Metabolism, Department of Internal Medicine, Seoul St. Mary’s Hospital, Seoul, 06591 Korea

## Abstract

Obesity is a well-known risk factor for type 2 diabetes, but few data exist on the association between weight changes and diabetes risk in non-obese subjects. This study aimed to investigate the effect of weight changes on the incidence of type 2 diabetes in Korea, using 51,405 non-diabetic subjects. Individuals who developed type 2 diabetes were more likely to be older and male, to have high body mass index (BMI), blood pressure, fasting blood glucose, and total cholesterol, to be current smokers and frequent drinkers, to be hypertensive and hyperlipidemic, and to have a family history of diabetes, compared to those without type 2 diabetes. Compared with the consistently non-obese group, there was a higher hazard ratio for incident diabetes (95% confidence interval) in subjects becoming obese [1.49 (1.26–1.77)] and remaining obese [2.56 (2.34–2.81)] after adjustment for confounding factors. Decreased BMI was significantly associated with lower risks for incident diabetes and the trends were more evident in the non-obese group. However, overall there was no significant association of increased BMI with incident diabetes. In conclusion, weight loss was significantly associated with lower risk for diabetes both in non-obese and obese Koreans, but particularly in the non-obese.

## Introduction

Type 2 diabetes is a chronic disease that is rapidly increasing in prevalence and has become a global health issue due to its serious long-term complications and the associated economic burden. The International Diabetes Federation reported that approximately 425 million individuals had type 2 diabetes worldwide in 2017, and this number was expected to reach 629 million by 2045^1^. Around 60% of the world’s diabetic population lives in Asia, and the increase in diabetes prevalence has been marked here, following recent economic development and over-nutrition. Type 2 diabetes in Asia is particularly characterized by onset at lower body weight and younger ages than in the Western population, leading to a higher risk of long-term consequences and mortality^2^.

The type 2 diabetes pandemic has been principally attributed to a global increase in obesity, a well-known risk factor for diabetes. Large randomized trials of lifestyle intervention have reported that weight reduction efficiently lowers the incidence of type 2 diabetes in prediabetic patients during the intervention^3–5^ and at follow-up^6–8^. Thus, the current guidelines recommend that subjects with prediabetes should increase their physical activity, with a goal of 7% weight loss, as a preventive strategy for diabetes^9^. However, over 60% of Asians with diabetes are non-obese, even if the Asian criteria are used to define obesity (BMI ≥ 25 kg/m^2^, which is a considerably lower limit than the 30 kg/m^2^ used generally)^10–12^. In addition, the Asia Cohort Consortium found a positive but rather weak association between BMI and diabetes prevalence compared to Western populations^13^, raising the possibility of ethnic differences in the efficacy of weight control for diabetes prevention. However, most studies have been conducted in Western countries with subjects of average BMI greater than 25 kg/m^2^ when prediabetic; therefore, few data exist regarding the efficacy of weight reduction for Asian individuals, particularly for those who are non-obese.

In this study, we aimed to prospectively investigate the association between weight changes and diabetes incidence in Korea and to compare the changes in risk between subjects who were initially non-obese and obese.

## Research Design and Methods

### Study population

Korea has a compulsory national health insurance system that covers all Korean residents, and the National Health Insurance Service (NHIS) has provided universal health coverage since 2000. The NHIS established the nationwide National Sample Cohort (NHIS-NSC), which contains public health information, such as the participants’ medical bill expenses claimed by medical service providers. A total of 1,025,340 subjects that were representative of the national population were randomly selected in 2002, which amounts to ~2.2% of the population, and were followed until 2013^14^. Proportionate stratified random sampling was conducted based on 1,476 strata. The data obtained for the cohort included insurance eligibility, medical care institutions consulted, medical treatments, and health screening data. The NHIS provides general health checkups and a cancer-screening program. All insured employees or self-employed persons aged 40 years and older, and their dependents can avail themselves of free health checkups at least biannually, and individuals above a certain age can undergo examinations for specific cancers at 10% of the usual cost.

From this cohort, we selected subjects who had received health examinations in 2002 and were re-examined in 2004 and 2006 and followed them until 2013 (mean 6.8 years, median 7.0 years). After excluding those who were diagnosed with diabetes mellitus prior to enrollment, 51,405 subjects were finally included and followed until the development of diabetes or the end of 2013. The patients who had developed diabetes during the study period were identified based on the prescription of antidiabetic medication under the World Health Organization (WHO) International Classification of Diseases (ICD)-10 codes E11-14 or fasting glucose level ≥126 mg/dL^15^.

### Determinants of disease and demographic factors

The body mass index (BMI) was calculated by dividing the subject’s weight by his/her height squared (kg/m^2^), obtained during regular medical checkup programs. Systolic and diastolic pressure was measured at the same time. Serum samples for measurement of fasting glucose, hemoglobin, and total cholesterol (TC) levels were obtained after an overnight fast prior to each examination. Detailed histories of smoking status, alcohol consumption and physical activity were obtained via a questionnaire. Statistical analyses were conducted using the simplified status classification of smoking (no, past, or current), alcohol consumption (No & <1/week, ≥1 time/week), and physical activity (no activity, ≤4 times/week, or ≥5 times/week). The subjects’ socioeconomic status was categorized into two groups based on income level, which was dichotomized at lower 20% (<20% vs. ≥20%). Hypertension was defined based on ICD-10 codes (I10–13, I15), the list of prescribed medicine, or blood pressure above 140/90 mmHg^15^. Hyperlipidemia was defined based on at least one of the following criteria: a claim under ICD-10 code (E78), the list of prescribed lipid lowering agents, or serum TC above 240 mg/dL^15^. Obesity was defined by a BMI of ≥25 kg/m^2^. The Institutional Review Board of The Catholic University of Korea approved the study protocol (No. SIRB-0E224-004) and informed consent was waived because of the anonymous nature of the data. This study was carried out according to the ethical principles of the Declaration of Helsinki.

### Statistical analysis

Statistical analyses were performed using SAS version 9.3 (SAS Institute, Cary, NC, USA). Continuous variables are expressed as mean ± standard deviation when normally distributed, or median (5–95% range) when the data were highly skewed. Continuous variables were compared using Student’s t-test or one-way analysis of variance (ANOVA), whereas categorical variables were compared using the χ^2^ test. Variables with skewed distributions were assessed as continuous variables following log transformation. Cox proportional hazards analysis was used to calculate the hazard ratio (HR) and 95% confidence interval (CI) for the association of BMI category with incident diabetes. *P* < 0.05 was considered statistically significant.

## Results

Of 51,405 participants included in 2002, 2,749 incident cases of diabetes were identified during the follow-up. As shown in Table 1, participants who developed diabetes were more likely to be older and male, to have higher BMI, systolic blood pressure (SBP), diastolic blood pressure (DBP), fasting blood glucose (FBG), and TC compared to those without diabetes. They tended to be current smokers and frequent drinkers (≥1 time/week) and to have lower rates of low income and higher rates of hypertension (HTN), hyperlipidemia, and a family history of diabetes. Factors associated with BMI changes are shown in Table 2. After 4 years, 10.2% of the initially non-obese group had become obese (the “becoming obese” group), whereas 18.2% of the initially obese group were no longer obese (the “slimming down” group). Subjects that had become obese tended to be men, to have higher SBP, DBP, FBG, and TC, to be current smokers and frequent drinkers, to take less exercise, and to have a more frequent family history of HTN, and were more likely to have diabetes than the subjects that had remained non-obese. Conversely, subjects that were no longer obese were more likely to be women, to have lower SBP, DBP, FBG, and TC, to be non-smokers, to do more exercise, and to have a less frequent family history of HTN, and were less likely to have diabetes than the subjects that had remained obese.

**Table 1:** Baseline characteristics classified according to subsequent onset of diabetes mellitus.

	New-onset diabetes mellitus		*P*
	No (n = 48656)	Yes (n = 2749)	
Age (years, %)			<0.001
20–39	22177 (45.6)	653 (23.8)	
40–64	24831 (51.0)	1874 (68.2)	
≥65	1648 (3.4)	222 (8.1)	
Male (%)	34002 (69.9)	2072 (75.4)	<0.001
BMI (kg/m^2^)			
2002	23.4 ± 2.9	25.4 ± 3.0	<0.001
2003^a^	23.5 ± 2.9	25.5 ± 3.2	<0.001
2004	23.5 ± 2.9	25.3 ± 3.1	<0.001
2005^b^	23.6 ± 2.8	25.5 ± 3.2	<0.001
2006	23.6 ± 2.9	25.4 ± 3.1	<0.001
SBP (mmHg)	122.7 ± 14.9	129.9 ± 15.7	<0.001
DBP (mmHg)	77.2 ± 10.1	81.4 ± 10.3	<0.001
FBG (mg/dL)	91.5 ± 14.1	114.5 ± 34.3	<0.001
TC (mg/dL)	194.2 ± 35.2	207.2 ± 38.7	<0.001
Smoking (%)			<0.001
No	27153 (63.6)	1442 (60.1)	0.002
Past	3238 (7.6)	189 (7.9)	
Current	12277 (28.8)	768 (32.0)	
Alcohol (%)			0.003
No & <1/week	32105 (68.1)	1748 (65.5)	
≥1 time/week	15016 (31.9)	921 (34.5)	
Exercise			<0.001
No	21769 (45.7)	1214 (45.4)	
≤4 times/week	22729 (47.7)	1210 (45.3)	
≥5 times/week	3163 (6.6)	248 (9.3)	
Low income (%)^c^	4026 (8.3)	311 (11.3)	<0.001
HTN (%)	9257 (19.0)	1091 (39.7)	<0.001
Dyslipidemia (%)	5296 (10.9)	604 (22.0)	<0.001
Family Hx. of HTN (%)	4393 (10.0)	270 (10.8)	0.154
Family Hx. of stroke (%)	2474 (5.6)	140 (5.6)	0.976
Family Hx. of CAD (%)	1232 (2.8)	62 (2.5)	0.383
Family Hx. of DM (%)	3241 (7.3)	278 (11.1)	<0.001
Family Hx. of cancer (%)	6164 (13.9)	324 (12.9)	0.182

Data are expressed as mean ± standard deviation, or number (percentage), unless otherwise indicated. BMI, body mass index; SBP, systolic blood pressure; DBP, diastolic blood pressure; FBG, fasting blood glucose; TC, total cholesterol; Hx, history; HTN, hypertension; CAD, coronary artery disease; DM, diabetes mellitus. ^a^n = 24512, ^b^n = 25427. ^c^Low income was defined as those bottom 20% of incomes.

**Table 2:** Factors associated with changes in BMI categories during the study.

2002	NonOB		*P1*	OB		*P2*	*P3*
2006	NonOB	OB		NonOB	OB		
n	32326	3682		2801	12596		
Age ≥ 65 yrs	1216 (3.8)	139 (3.8)	0.968	111 (4.0)	404 (3.2)	0.044	0.028
Female (%)	11090 (34.3)	815 (22.1)	<0.001	797 (28.5)	2629 (20.9)	<0.001	<0.001
BMI (kg/m^2^)	21.8 ± 1.9	24.0 ± 1.0	<0.001	25.9 ± 1.5	27.2 ± 1.8	<0.001	<0.001
SBP (mmHg)	120.6 ± 14.6	126.0 ± 14.5	<0.001	124.2 ± 14.7	128.3 ± 15.0	<0.001	<0.001
DBP (mmHg)	75.9 ± 9.9	79.2 ± 9.9	<0.001	77.9 ± 9.9	80.9 ± 10.2	<0.001	<0.001
FBG (mg/dL)	91.3 ± 15.3	93.6 ± 15.4	<0.001	94.3 ± 21.5	95.8 ± 18.7	<0.001	<0.001
TC (mg/dL)	190.6 ± 34.4	202.0 ± 36.5	<0.001	195.3 ± 36.4	203.6 ± 35.7	<0.001	<0.001
Smoking (%)			<0.001			<0.001	<0.001
No	18664 (65.3)	1873 (59.3)		1635 (66.0)	6423 (59.1)		
Past	1949 (6.8)	317 (10.0)		204 (8.2)	958 (8.8)		
Current	7958 (27.9)	970 (30.7)		638 (25.8)	3480 (32.0)		
Alcohol (%)			<0.001			<0.001	<0.001
No & <1/week	22057 (70.5)	2252(63.3)		1866 (68.8)	7678 (62.8)		
≥1/week	9231 (29.5)	1308 (36.7)		848 (31.3)	4550 (37.2)		
Exercise			<0.001			<0.001	<0.001
No	15242 (48.2)	1606 (44.5)		1051 (38.2)	5084 (41.2)		
≤4/week	14430 (45.6)	1795 (49.7)		1372 (49.9)	6342 (51.4)		
≥5/week	1958 (6.2)	209 (5.8)		329 (12.0)	915 (7.4)		
Low income (%)	2679 (8.3)	289 (7.9)	0.359	271 (9.7)	1098 (8.7)	0.107	<0.001
Family Hx. of HTN (%)	2707 (9.3)	369 (11.0)	0.001	279 (10.9)	1310 (11.5)	0.407	<0.001
Family Hx. of stroke (%)	1612 (5.5)	175 (5.2)	0.506	137 (5.4)	690 (6.0)	0.187	0.117
Family Hx. of CAD (%)	812 (2.8)	97 (2.9)	0.681	72 (2.8)	314 (2.8)	0.866	0.975
Family Hx. of DM (%)	2125 (7.2)	241 (7.2)	0.897	218 (8.5)	935 (8.2)	0.589	0.002
Family Hx. of cancer (%)	4175 (14.2)	432 (12.9)	0.042	343 (13.3)	1538 (13.4)	0.936	0.055
Incident diabetes (%)	1075 (3.3)	192 (5.2)	<0.001	207 (7.4)	1275 (10.1)	<0.001	<0.001

**Figure 1:**
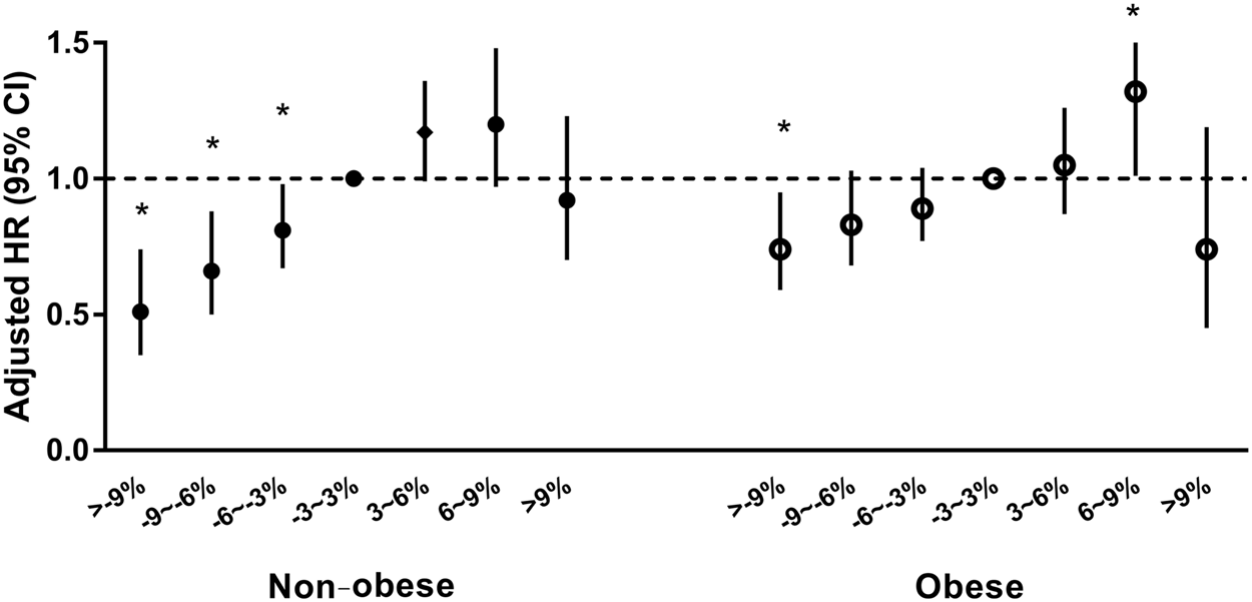
Cox regression to evaluate the HR and 95% CI for incident diabetes by BMI changes compared to those with −3~3% changes. The multivariate model was adjusted for age, sex, current smoking, alcohol drinking, exercise, income, hypertension, dyslipidemia and a family history of diabetes. *P < 0.05.

The HRs for changes in BMI status for incident diabetes were also calculated (Table 3). Compared with the group that had remained non-obese, there were higher HRs (95% CI) for incident diabetes in “becoming obese” [1.49 (1.26–1.77)], “slimming down” [1.86 (1.58–2.20)], and “still obese” subjects [2.56 (2.34–2.81)] in the analysis of 4-year weight change, after adjusting for age, sex, current smoking, alcohol drinking, exercise, income, HTN, dyslipidemia, and a family history of diabetes. When the data were classified into seven groups based on BMI, reduced BMI was significantly associated with lower risks for incident diabetes (Table 4; Fig. 1). These trends were more evident in the non-obese group than in the obese group; statistical significance was observed from BMI changes (−3%~−6%) among the non-obese whereas BMI changes >−9% showed significant association with the incident diabetes among the obese. However, there was no significant association between weight gain and incident diabetes except for a positive association in obese subjects whose BMI increased by 6–9%.

**Table 3:** Association between changes in BMI categories over 2 or 4 years and incident type 2 diabetes.

BMI category			Incidence rate^a^	HR (95% CI)		
				Unadjusted	Age-, sex-adjusted	MV^b^-adjusted
**2002** → **2006**						
NonOB	NonOB	still non-obese	4.82	1	1	1
	OB	becoming obese	7.62	1.58 (1.36–1.84)	1.55 (1.33–1.81)	1.49 (1.26–1.77)
OB	NonOB	slimming down	10.95	2.27 (1.96–2.64)	2.01 (1.73–2.33)	1.86 (1.58–2.20)
	OB	still obese	15.18	3.15 (2.91–3.42)	2.91 (2.69–3.16)	2.56 (2.34–2.81)
	*P*			<0.001	<0.001	<0.001
**2002 → 2004**						
NonOB	NonOB	still non-obese	4.80	1	1	1
	OB	becoming obese	8.59	1.78 (1.52–2.10)	1.73 (1.47–2.03)	1.74 (1.46–2.08)
OB	NonOB	slimming down	10.60	2.22 (1.90–2.58)	1.94 (1.66–2.26)	1.71 (1.43–2.04)
	OB	still obese	15.21	3.17 (2.93–3.44)	2.93 (2.70–3.18)	2.62 (2.39–2.87)
	*P*			<0.001	<0.001	<0.001

^a^Per 1000 person-years. ^b^MV, multivariate includes age, sex, current smoking, alcohol drinking, exercise, income, hypertension, dyslipidemia, and a family history of diabetes.

**Table 4:** Hazard ratios (95% confidence intervals) for new-onset diabetes according to changes in BMI.

Group	BMI changes	2002 → 2006 Incidence rate^a^	HR (95% CI)			2002 → 2004 Incidence rate^a^	HR (95% CI)		
			Unadjusted	Age-, sex-adjusted	MV^b^-adjusted		Unadjusted	Age-, sex-adjusted	MV^b^-adjusted
**Non-OB**	>−9%	2.3	**0.43 (0.30–0.60)**	**0.52 (0.37–0.74)**	**0.51 (0.35–0.74)**	3.4	**0.60 (0.41–0.87)**	**0.68 (0.47–0.99)**	0.73 (0.50–1.07)
	−9–−6%	3.2	**0.59 (0.46–0.77)**	**0.68 (0.52–0.88)**	**0.66 (0.50–0.88)**	3.0	**0.53 (0.39–0.72)**	**0.58 (0.43–0.79)**	**0.61 (0.44–0.84)**
	−6 – −3%	3.9	**0.71 (0.60–0.85)**	**0.80 (0.67–0.96)**	**0.81 (0.67–0.98)**	4.8	**0.85 (0.72–1.00)**	0.94 (0.80–1.10)	0.89 (0.75–1.07)
	−3–3%	5.5	1	1	1	5.6	1	1	1
	3–6%	7.2	**1.31 (1.13–1.52)**	**1.16 (1.00–1.34)**	1.17 (0.99–1.36)	5	0.89 (0.76–1.05)	**0.80 (0.68–0.95)**	0.78 (0.65–0.92)
	6–9%	8.1	**1.46 (1.20–1.78)**	**1.26 (1.03–1.53)**	1.20 (0.97–1.48)	7.5	**1.34 (1.07–1.66)**	1.19 (0.95–1.48)	1.13 (0.90–1.43)
	>9%	6.4	1.16 (0.89–1.51)	0.93 (0.71–1.22)	0.92 (0.70–1.23)	7.3	1.30 (0.97–1.76)	1.08 (0.80–1.45)	1.04 (0.75–1.43)
	*P*		<0.001	<0.001	<0.001	3.4	<0.001	<0.001	0.002
**OB**	>−9%	8.9	**0.61 (0.48–0.76)**	**0.73 (0.59–0.92)**	**0.74 (0.59–0.95)**	9.9	**0.72 (0.53–0.99)**	0.84 (0.61–1.15)	0.77 (0.54–1.09)
	−9–−6%	10.7	**0.73 (0.60–0.89)**	**0.81 (0.67–0.99)**	0.83 (0.68–1.03)	11.0	0.80 (0.63–1.01)	0.91 (0.72–1.15)	0.89 (0.69–1.14)
	−6–−3%	12.2	**0.83 (0.72–0.96)**	0.88 (0.76–1.01)	0.89 (0.77–1.04)	13.8	1.01 (0.87–1.16)	1.06 (0.92–1.23)	1.01 (0.86–1.19)
	−3–3%	14.6	1	1	1	13.7	1	1	1
	3–6%	15.5	1.06 (0.89–1.26)	1.02 (0.86–1.21)	1.05 (0.87–1.26)	13.5	0.98 (0.83–1.16)	0.95 (0.80–1.12)	0.93 (0.78–1.11)
	6–9%	18.7	1.28 (0.99–1.65)	1.25 (0.97–1.61)	**1.32 (1.01–1.73)**	12.4	0.90 (0.68–1.19)	0.88 (0.66–1.16)	0.81 (0.60–1.11)
	>9%	13.5	0.92 (0.61–1.40)	0.93 (0.61–1.40)	0.74 (0.45–1.19)	18.1	1.32 (0.96–1.80)	1.30 (0.95–1.78)	1.35 (0.98–1.87)
	*P*		<0.001	<0.001	<0.001		0.074	0.328	0.466

^a^Per 1000 person-years. ^b^MV includes age, sex, current smoking, alcohol drinking, exercise, income, hypertension, dyslipidemia, and a family history of diabetes.

## Discussion

The present study showed that weight loss was significantly associated with a lower incidence of type 2 diabetes than weight maintenance. In addition, even a slight loss in BMI (−3%~−6%) by non-obese subjects was associated with decreased incidence of type 2 diabetes versus obese subjects. When the association of change in obesity status and the incidence of type 2 diabetes was considered as a categorical variable, “slimming down” group was significantly associated with decreased risk of diabetes development compared to the “still obese” group, but the hazard ratio of diabetes incidence was still higher than for the “becoming obese” subjects who were lean at baseline. Conversely, there was a trend for weight gain to induce the development of diabetes; “becoming obese” and “still obese” subjects had greater risk of incident diabetes compared to “still non-obese” subjects. However, analysis of changes in subdivided BMI did not show clear association between increased BMI and new-onset diabetes,

Large-scale clinical trials found that lifestyle intervention targeting weight loss efficiently reduces diabetes incidence in subjects with prediabetes: a 58% reduction over 3.2 years was shown in the Finnish Diabetes Prevention Study^3^, a 58% reduction over 2.8 years in the U.S. Diabetes Prevention Program (DPP)^4^ and a 46.0% reduction over 6 years in the Da Qing study^5^. Follow-up of these studies revealed persistently lowered type 2 diabetes incidence, ranging from 34% to 43% between 7 and 20 years^6–8^. The role of pharmacological intervention has not been determined, but metformin, the most intensively studied drug, is reported to reduce the risk of developing type 2 diabetes in the DPP by 30%, which occurred alongside an average 1.7 kg weight loss, which explained 64% of its beneficial effects on diabetes risk^16^. Although observational studies have shown inconsistent results^17–22^, data from intervention studies conducted in at-risk populations, such as those with prediabetes or the obese/overweight^3–5,23^, have been more consistent in their outcomes. Consistent with our results, several studies found significant associations between weight loss and decreased diabetes incidence in the general population^17,18^, questioning proper candidates for lifestyle intervention targeting weight loss, although controversial data exist^19–22^. In addition, our results suggest that weight reduction may be more effective in lowering diabetes risk in non-obese than obese individuals.

In this study, we found that weight gain was more likely to occur in men, to be associated with unfavorable metabolic profiles, including higher SBP, DBP, FBG, and TC, and to be associated with a history of HTN and an unhealthy lifestyle, including smoking, alcohol consumption, and less frequent exercise, than stable weight. Our data suggest that subjects who gained weight may be more insulin resistant than those who maintained the same degree of obesity. This finding is consistent with the study by Meigs *et al*.^24^, which found that metabolic risk was a separate and more important risk factor for diabetes development than obesity *per se*. However, the present study found no association between weight gain and diabetes incidence, which is not consistent with other studies^25^, although examination of the effects of changes in obesity categorization on diabetes incidence indicates the same trend. This discrepancy may be the result of the relatively short period of this study, giving insufficient time for the effects of cumulative weight gain to manifest. In addition, Wei *et al*.^26^ reported that adiposity was more strongly associated with diabetes risk in young adulthood than in middle-aged adults, and speculated that older adults may not be affected by the relatively small increase in risk of diabetes following weight gain, because they are already at higher risk. Thus, an alternative explanation for the lack of association between weight gain and diabetes incidence in this study may be that we included middle-aged and elderly subjects.

In the present study, smokers tended to develop diabetes more frequently, which is consistent with the findings of a meta-analysis, which showed that current smokers had a 44% higher risk of developing type 2 diabetes^27^. A number of studies show beneficial effects of light to moderate alcohol drinking in diabetes prevention^28^, but frequent drinkers were more likely to develop diabetes in this study. We speculate that this discrepancy may be related to lack of information on the amounts of alcohol consumed, which prevents a separate analysis of light to moderate drinking, and to confounding factors, such as concurrent use of tobacco in alcohol drinkers, which is commonly found in Asian subjects^28^. Unexpectedly, exercise seemed to be associated with new-onset diabetes in a simple analysis. However, the observed association between exercise and new-onset diabetes was likely to be caused by confounding bias of obesity, a well-established risk factor for diabetes; there was positive correlation between obesity and physical activity as shown in a previous nationwide cohort^29^ and ours.

### Mechanisms

The mechanism underlying the diabetes preventive ability of weight loss may be related to increased insulin sensitivity, thus delaying or preventing beta-cell failure. The reason why weight reduction has more prominent effect on lower risks for T2DM incidence in non-obese subjects and whether there are ethnic differences^30^.

### Clinical implications

Based on our data, lifestyle changes aimed at weight reduction can be recommended. For non-obese individuals, targeting a 3% reduction in BMI could be effective, and, for obese individuals, a reduction of more than 9% should be recommended over 4 years. More long-term studies should be undertaken to establish whether more ambitious weight reduction goals might be needed for obese Koreans and whether weight loss, even to a small extent, might be beneficial for non-obese individuals. However, caution should be taken in interpreting our data because the positive association between weight loss and lower incidence of diabetes does not necessarily mean that lean subjects are equally recommended to lose weight for diabetes prevention. In addition, although there was significant association between weight loss and lower incidence of diabetes in a dose-dependent manner, it is questionable that a larger reduction of BMI (i.e., ≥−9%) may provide the greatest benefit to lean subjects. Considering previous studies reporting that normal weight subjects with type 2 diabetes had a poorer prognosis following cardiovascular disease events and higher mortality rates than overweight or obese subjects^31^, further longitudinal studies are needed to determine the effect of weight loss on reduced diabetes risk and desirable weight loss target in non-obese subjects without raising health concerns such as cardiovascular disease.

### Limitations

The strengths of the present study include the large sample size. Second, it was a prospective cohort study, which ensured a thorough follow-up. In addition, the database used was stable, as it is maintained by the government or public institutions involved in providing national health information^32^. Third, the data contain lifestyle and demographic characteristics, including smoking, alcohol consumption, physical activity, and income status, facilitating control regarding potential confounding factors.

However, the study has several limitations. First, there is no information on whether the weight loss was intentional, or how it was achieved, either by diet or by exercise. Furthermore, we could not exclude subjects with serious medical conditions from the weight loss group. However, as this was a large cohort and the stable weight group tended to be more regular exercisers than the weight gain group, it is unlikely that subjects with severe medical conditions would have biased the results. Second, the study period was relatively short, meaning that the results may have been different with a longer follow-up. Third, our data cannot be generalized to other ethnicities, as this study included only Korean subjects. Fourth, diabetes incidence could be underestimated. As the presence of diabetes was defined by based on the prescription of antidiabetic medication under ICD-10 codes E11–14 or fasting glucose level ≥ 126 mg/subjects with undiagnosed diabetes or those who did not visit a hospital during the study period could be omitted from these data. Fifth, there are possibility of selection bias. As subjects were enrolled, among adults who had received health examination and follow-up after two or four years, our cohort may include healthier and/or more health conscious subjects than general population. Lastly, there are no reports on quantified validation for the questionnaire variables such as smoking status, alcohol use, and physical activity. In conclusion, weight changes are closely associated with diabetes incidence in the Korean population. In particular, weight reduction was more strongly associated with reduced risk of diabetes in non-obese than in obese subjects. Further long-term studies are needed to validate the usefulness of weight reduction for the prevention of diabetes in the mostly non-obese Korean population.
